# Tracking the Impact of Excisional Cervical Treatment on the Cervix using Biospectroscopy

**DOI:** 10.1038/srep38921

**Published:** 2016-12-15

**Authors:** Diane E. Halliwell, Maria Kyrgiou, Anita Mitra, Ilkka Kalliala, Evangelos Paraskevaidis, Georgios Theophilou, Pierre L. Martin-Hirsch, Francis L. Martin

**Affiliations:** 1 Centre for Biophotonics, LEC, Lancaster University, Lancaster, UK; 2 Institute of Reproductive and Developmental Biology, Department of Surgery & Cancer, Faculty of Medicine, Imperial College, London, UK; 3 West London Gynaecological Cancer Centre, Imperial College NHS Healthcare, London, UK; 4 Department of Obstetrics and Gynaecology, University of Ioannina, Ioannina, Greece; 5 St James Hospital, Leeds, West Yorkshire, UK; 6 Department of Obstetrics and Gynaecology, Lancashire Teaching Hospitals NHS Trust Foundation, Preston, UK; 7 School of Pharmacy and Biomedical Sciences, University of Central Lancashire, Preston, UK

## Abstract

Local excisional treatment for cervical intra-epithelial neoplasia (CIN) is linked to significant adverse sequelae including preterm birth, with cone depth and radicality of treatment correlating to the frequency and severity of adverse events. Attenuated total reflection Fourier-transform infrared (ATR-FTIR) spectroscopy can detect underlying cervical disease more accurately than conventional cytology. The chemical profile of cells pre- and post-treatment may differ as a result of altered biochemical processes due to excision, or treatment of the disease. Since pre-treatment cervical length varies amongst women, the percentage of cervix excised may correlate more accurately to risk than absolute dimensions. We show that treatment for CIN significantly alters the biochemistry of the cervix, compared with women who have not had treatment; this is due to the removal of cervical tissue rather than the removal of the disease. However, the spectra do not seem to correlate to the cone depth or proportion of cervical length excised. Future research should aim to explore the impact of treatment in a larger cohort.

Persistent infection with a high-risk oncogenic human papillomavirus (HPV) subtypes is necessary for the development of invasive cervical cancer and its precancerous precursor (cervical intra-epithelial neoplasia; CIN)^1^. Over 80% of sexually active women will have been infected with HPV at some point by the age of 50^2^, although more than 90% of these infections are transient, being cleared by an incompletely understood immune response within 6–18 months^3^. Even when low-grade pre-invasive disease (CIN1) develops, the majority of these lesions regress spontaneously back to normal, particularly in young women^4^, while CIN2 or worse is considered the histological cut-off to proceed to treatment.

Local conservative treatment for CIN removes or ablates a cone-shaped part of the cervix containing the precancerous cells. The choice of treatment technique varies across Europe and beyond. In some countries, knife excision (cold knife conisation; CKC) is still regularly performed; in others, ablation (LA) or conisation (LC) with the laser beam is common practice. Outpatient large loop excision of the transformation zone (LLETZ) is the preferred treatment in most settings, as this offers many advantages including low cost, high success rate and ease of use^5^. Furthermore, excisional techniques allow the histological assessment of the tissue removed, while ablative techniques preclude this and demand a colposcopically-directed biopsy at a separate visit.

Local conservative treatments for pre-invasive cervical lesions are highly effective (90–95%) in preventing recurrent cervical disease and future invasion^6^. The majority of women clear the infection after local treatment, although those that remain positive for HPV are at higher risk of recurrence^7^. The mean age of women undergoing treatment for pre-invasive cervical disease is similar to the age of women having their first child. There is mounting evidence that excisional treatment is associated with a significant risk to future pregnancies, including preterm birth and mid-trimester loss^8^,^9^,^10^,^11^. It has been postulated that removal of part of the cervix possibly leads to acquired mechanical weakness as a result of collagen disruption. Furthermore, endocervical glands produce the mucus plug during pregnancy which has antimicrobial properties against Gram positive and negative bacteria; thus their removal may damage the host defense mechanism against ascending infections when pregnant^10^,^11^.

The cone depth and radicality of treatment have been found to be directly correlated to the frequency and severity of the adverse events^8^,^12^. Given that the pre-treatment cervix length and dimensions vary amongst women, it is biologically plausible that the proportion (percentage) of the cervix excised may correlate more accurately to the chances of adverse sequelae than the absolute dimensions, and that there may be a cut-off for the proportion of excision that signifies women at risk^13^,^14^,^15^.

Attenuated total reflection Fourier-transform infrared (ATR-FTIR) spectroscopy has shown potential in the field of cancer screening, being an inexpensive but robust technique. Previous research demonstrated that the technique is able to segregate grades of cervical cytology^16^,^17^, classify cervical cytology based on HPV infection with low- or high-risk types^18^, and diagnose underlying disease more accurately that conventional cytology screening^19^. Infrared (IR) spectroscopy exploits the molecular vibrations of biologically active molecules that are created by dipole moments as a result of being flooded with IR light. The ‘biochemical fingerprint’ produced for normal or dysplastic cells, and cells before and after treatment may differ based on changes in availability of lipids, proteins, carbohydrates and so on. This may arise from altered biochemical processes as a result of treatment of the disease.

The aim of this study was to assess the impact that local treatment may have on the cervix by comparing the spectral absorbance within the ‘fingerprint region’ of cervical cells before and after treatment, and to evaluate how these changes may be affected by the absolute cone dimensions or the proportion (percentage) of cervix excised.

## Results

**Figure 1:**
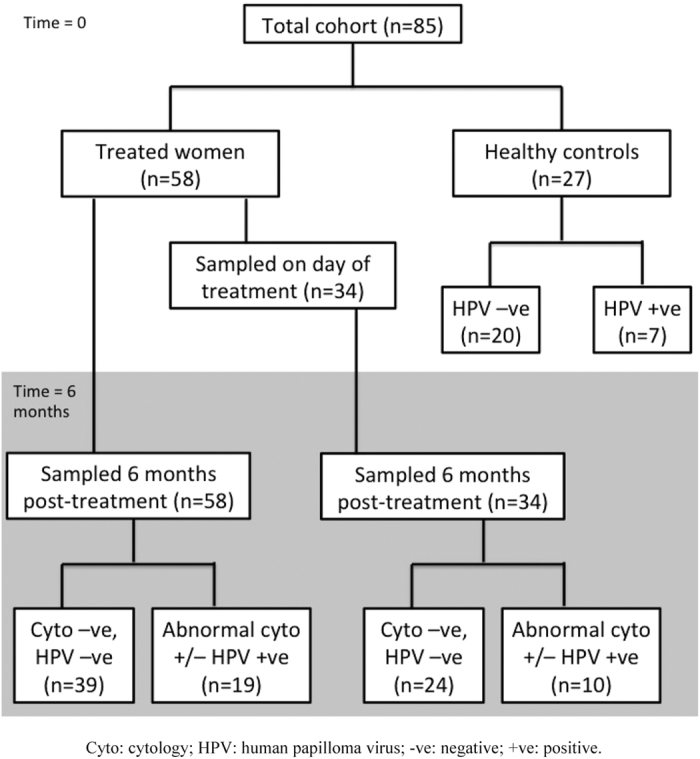
Flowchart of the included population and different comparison groups.

We enrolled a total of 58 women planned to undergo cervical treatment into the study and 27 healthy controls (cytology negative); 20 of these were also HPV test negative. Out of the 58 treated women, 34 women had paired samples before and after treatment and 58 had at least post-treatment samples; 39 out of these 58 women had normal results post-treatment (cytology and HPV DNA test negative). The depth of the excised cone was available for all 58 women who underwent treatment and the proportion of cervical length removed available for 53 of these (91%). The different clinical groups are described in Fig. 1.

Out of the 34 women with paired samples before and after treatment (Comparison 1), 5 samples pre- and 1 sample post-treatment did not provide reliable spectra, allowing 29 and 33 samples for analysis, respectively. There were no major differences between the pre- and post-treatment samples. The rate of women who had sexual intercourse less than 48 hours from the sample collection was 90% and 85%, at pre- and post-treatment sampling, respectively (p = 0.71). None of the patients reported the use of vaginal douching.

Thirty-nine out of 58 women that had at least a post-treatment sample were normal with negative cytology and a negative HPV test post-treatment. The characteristics of this subgroup (n = 39) were largely similar to the normal healthy controls (cytology/HPV test negative) that have had no local cervical treatment (n = 20) (Comparison 2). The mean age (SD) for treated and control groups was 30.8 years (4.5), and 30.6 years (4.3), respectively. The rate of women who were current smokers was significantly greater in the treated group (31%), compared with controls (5%); (p = 0.04), as was the proportion of women taking combined oral contraceptives (51% *vs*. 15% respectively, p = 0.01). Fewer controls were of Caucasian race (55% *vs*. 77%), and a greater proportion of women in the treated group had been sampled in the luteal phase (54%) compared with controls (30%), although neither of these differences however were statistically significant. The proportion of women reporting recent intercourse (25% *vs*. 13%), with bacterial vaginosis diagnoses on high-vaginal swabs (10% *vs*. 8%) and vaginal pH was also comparable between the control and treated groups.

A total of 58 women had data on the cone depth and proportion of depth excised; of these 5 were lacking data on the percentage of excision but had data on the depth. Mean depth (SD) of excision for the 58 women was 10.41 mm (3.8 mm); (range 2–17 mm), and the mean proportion of excision (SD) in the 53 women for whom this data was available was 29.13% (10.0%); (range 2–56%). Ten of the 58 treated women (17%) had involvement of the cone margins at histopathological analysis; 2/58 (3%) with HSIL and 8/58 (14%) with LSIL, however, only two of these 10 patients (3%) had abnormal cytology at 6 month follow-up. The characteristics of this group were overall similar to healthy controls with normal cytology irrespective of HPV status (n = 27) (Comparison 3) (Table 1 and Supplementary Table 2).

Once again the proportion of women using combined oral contraceptive pills was significantly greater in treated women (48% *vs*. 15%, p = 0.003), and a higher percentage of women in the treated group had been sampled in the luteal phase (50%) compared with controls (26%), which did not reach statistical significance. The groups were otherwise similar with respect to age, ethnicity, smoking, parity, recent intercourse, vaginal pH, bacterial vaginosis and HPV DNA status.

### Treatment for CIN significantly changes the biochemical fingerprint in the cervix, compared with women with pre-invasive disease who have not had treatment

**Figure 2:**
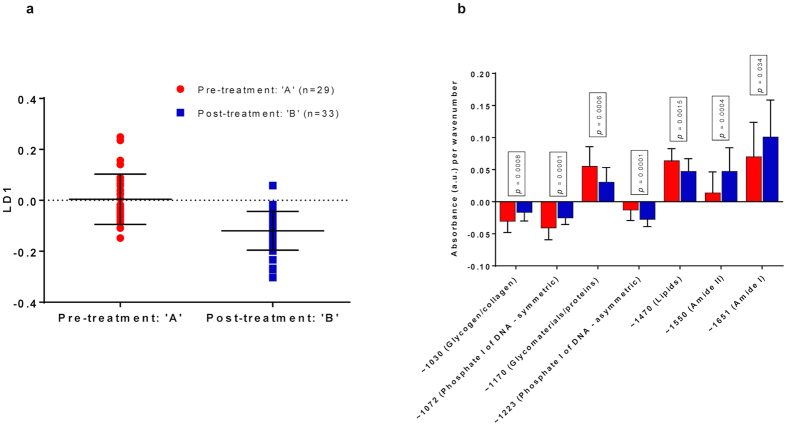
PCA-LDA scores plot of ATR-FTIR spectra with regards to LD1: Pre- vs Post-treatment (**a**) together with absorbance per wavenumber (**b**). The paired samples pre- and post-treatment were significantly different along LD1 (Mean/SD (**a**): 0.004/0.10 for ‘A’; −0.12/0.08 for ‘B’; p < 0.0001, 95% CI = −0.17 to −0.08). Absorbance associated with lipids, glycomaterials and proteins was shown to have a significant positive rate of change for the pre-treatment group compared with the post-treatment group, indicating their higher bioavailability. Similarly, absorbance associated Amide I and Amide II was shown to have a significant positive rate of change for the post-treatment group compared within the pre-treatment group. Absorbance associated with glycogen, collagen and symmetric phosphate of DNA was shown to have a significant negative rate of change for the pre-treatment group compared with the post-treatment group, suggesting lower bioavailability. Similarly, absorbance associated asymmetric phosphate for DNA was shown to have a significant negative rate of change for the post-treatment group compared with the pre-treatment group (**b**). ATR-FTIR: Attenuated total reflection Fourier-transform Infrared; CI: Confidence interval; LD1: Linear Discriminant 1; PCA-LDA: Principal Component Analysis coupled to Linear Discriminant Analysis; SD: Standard deviation.

When we compared the absorbance spectra before and after local excisional cervical treatment, we detected a statistically significant difference between the pre- and post-treatment paired samples (p < 0.0001; 95 CI = −0.17 to −0.08; Fig. 2a). A significant positive rate of change was found for absorbance associated with lipids (p = 0.0015), and for glycomaterials/proteins (p = 0.0006) for the pre-treatment samples as compared to the post-treatment samples, indicating higher bioavailability in the former group (Fig. 2b). A significant positive rate of change was found for absorbance associated with proteins featuring Amide I (p = 0.034) and Amide II (p = 0.0004) type bonding for post-treatment samples as compared the pre-treatment ones, indicating higher bioavailability of polypeptides in the post-treatment group. A significant negative rate of change was detected for absorbance associated with glycogen/collagen (p = 0.0008) and symmetric phosphate of DNA (p = 0.0001) in the pre- as opposed to the post-treatment group, signifying lower bioavailability in the pre-treatment group. Similarly, absorbance associated asymmetric phosphate for DNA was shown to have a significant negative rate of change for post- as opposed to pre-treatment samples (p = 0.0001); (Fig. 2b). The steps for pre-processing, multivariate analysis and extraction of wavenumbers are summarized in Supplementary Figures 1, 2 and 3.

### Changes in the biochemical fingerprint are due to excision of cervical tissue rather than removal of disease

**Figure 3:**
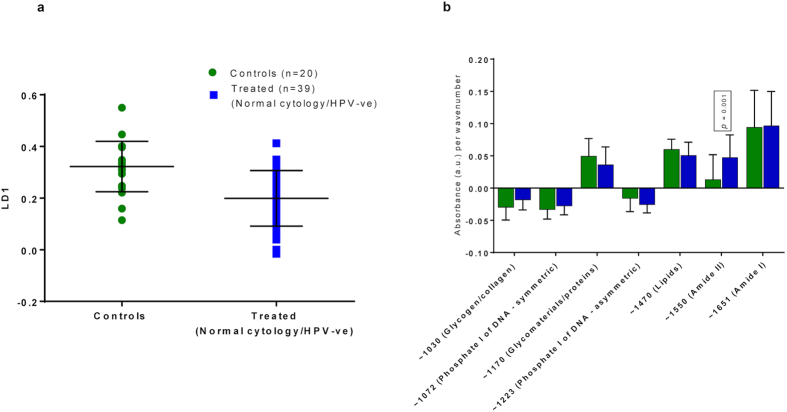
PCA-LDA scores plot of ATR-FTIR spectra with regards to LD1: controls vs treated (normal cytology and HPV –ve); (**a**) together with absorbance per wavenumber (**b**). The 2 groups were significantly different along LD1 (Mean/SD (**a**): 0.32/0.10 for Controls; 0.20/0.11 for Treated; p < 0.0001, 95% CI = −0.18 to −0.07). These results evidence that the difference in LD1 was due to the impact of treatment. Absorbance associated with Amide II was shown to have a significant positive rate of change for the treated group compared with controls, indicating higher bioavailability (**b**). No other significant changes were detected. ATR-FTIR: Attenuated total reflection Fourier-transform Infrared; CI: Confidence interval; LD1: Linear Discriminant 1; -ve: Negative; PCA-LDA: Principal Component Analysis coupled to Linear Discriminant Analysis; +ve: Positive; SD: Standard deviation.

In order to explore whether the observed differences before and after treatment were attributed to the treatment rather than the removal of a disease, we performed a subgroup analysis comparing all women that had at least one post-treatment sample with negative cytology and HPV DNA test (n = 39) versus healthy controls negative for cytology and HPV (n = 20). We found that the difference in the spectra post-treatment remained significant, evidencing that the difference observed before and after treatment was due to treatment intervention rather than the treatment of the disease (p < 0.0001; 95% CI = −0.18 to −0.07; Fig. 3a). A significant positive rate of change was found for absorbance associated with proteins featuring Amide II bonding (p = 0.001) in the treated group compared with controls; no other significant changes were detected (Fig. 3b).

### Spectra do not seem to correlate to the cone depth or proportion of cervical length excised

**Figure 4:**
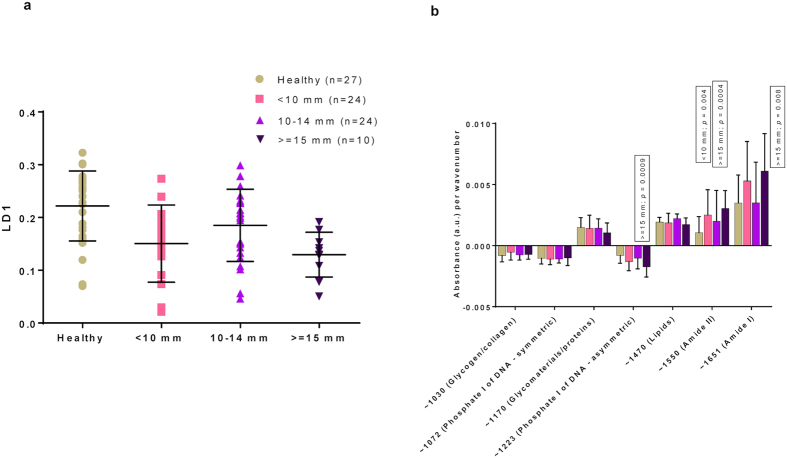
PCA-LDA scores plot of ATR-FTIR spectra with regards to LD1: Healthy Cervix *vs*. Cone Depth (**a**) together with absorbance per wavenumber (**b**). Mean/SD (**a**) for each group was: 0.22/0.07 for healthy cervix; 0.15/0.07 for <10 mm; 0.19/0.07 for 10–14 mm; 0.13/0.04 for ≥15 mm. A significant difference along LD1 was detected for healthy cervix *vs*. <10 mm (p = 0.0008; 95 CI = 0.03 to 0.12); and for healthy cervix *vs*. ≥15 mm (p = 0.001; 95 CI = 0.03 to 0.15). No significant difference along LD1 was detected for healthy cervix *vs*. 10–14 mm (p = 0.13; 95 CI = −0.01 to 0.08). Absorbance associated with Amide II was shown to have a significant positive rate of change for <10 mm group compared with healthy cervix, indicating higher bioavailability (**b**). Similarly, absorbance associated with Amide I and II were shown to have a significant positive rate of change for ≥15 mm compared with healthy cervix, whilst absorbance associated with DNA was shown to have a negative rate of change, indicating lower bioavailability. ATR-FTIR: Attenuated total reflection Fourier-transform Infrared; CI: Confidence interval; LD1: Linear Discriminant 1; PCA-LDA: Principal Component Analysis coupled to Linear Discriminant Analysis; SD: Standard deviation.

We further assessed whether the observed difference in the absorbance spectra correlated to the depth of the cone and the proportion of the depth excised in the pre-specified treated groups, and compared these to healthy controls that were cytology negative irrespective of HPV status (n = 27). We found overall that the spectra of treated women were different to the spectra of healthy controls but this did not seem to correlate to the cone depth and proportion of cervical length excised. More specifically, we detected statistically significant differences in the spectra when the healthy samples was compared to samples from treated patients with a cone depth of <10 mm (p = 0.0008; 95% CI = 0.03 to 0.12), a cone depth of ≥15 mm (p = 0.001; 95% CI = 0.03 to 0.15), but not for a cone depth between 10–14 mm (Fig. 4a). A significant positive rate of change was observed for absorbance associated with proteins featuring Amide II bonding for both the <10 mm and ≥15 mm groups compared to healthy samples (p = 0.004, p = 0.0004, respectively). Cone depth ≥15 mm also had a significant positive rate of change for absorbance associated with polypeptides featuring Amide I bonding (p = 0.008), and a significantly negative rate of change for asymmetric phosphate of DNA (p = 0.0009) as compared to healthy controls (Fig. 4b).

**Figure 5:**
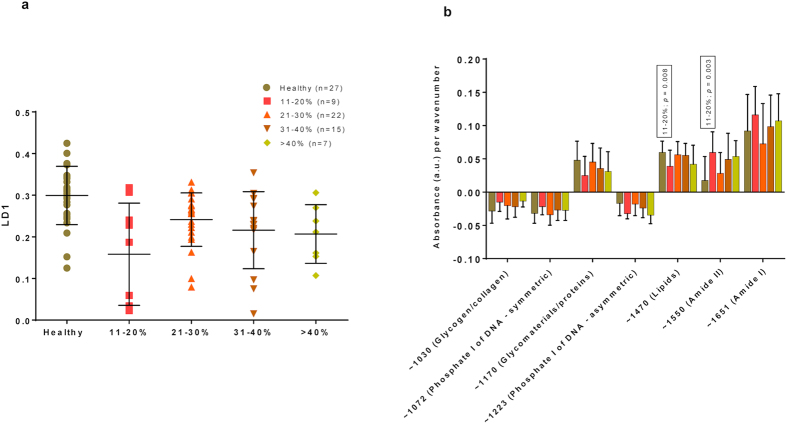
PCA-LDA scores plot of ATR-FTIR spectra with regards to LD1: Healthy Cervix *vs*. Percentage Excision (**a**) together with absorbance per wavenumber (**b**). Mean/SD (**a**) for each group was: 0.30/0.07 for healthy cervix; 0.16/0.12 for 11–20%; 0.24/0.06 for 21–30%; 0.22/0.09 for 31–40%; 0.21/0.07 for >40%. A significant difference along LD1 was detected for healthy cervix *vs*. 11–20% (p = 0.002; mean rank difference: 31.11); for healthy cervix *vs*. 21–30% (p = 0.03; mean rank difference: 17.85); for healthy cervix *vs*. 31–40% (p = 0.007; mean rank difference 23.33; and for healthy cervix *vs*. >40% (p = 0.023; mean rank difference: 27.24). Absorbance associated with lipids and Amide II was shown to have a significant positive rate of change for 11–20% compared with healthy cervix, indicating higher bioavailability (**b**). No other significant differences were detected for healthy cervix *vs*. all other groups. ATR-FTIR: Attenuated total reflection Fourier-transform Infrared; CI: Confidence interval; LD1: Linear Discriminant 1; PCA-LDA: Principal Component Analysis coupled to Linear Discriminant Analysis; SD: Standard deviation.

The results were similar when we correlated the spectra to the percentage of excision. The group of women that had <10% of their cervix excised included only 2 patients and was therefore excluded from the analysis. For all the remaining groups, we detected significant differences as compared with healthy cervix [11–20% (p = 0.002, mean rank difference = 31.11); 21–30% (p = 0.03, mean rank difference = 17.85); 31–40% (p = 0.007, mean rank difference = 23.33); and for >40% (p = 0.023, mean rank difference = 27.24)] (Fig. 5a). A significant positive rate of change in absorbance associated with biomarkers was found only for 11–20% compared with healthy cervix (lipids: p = 0.008) and polypeptides featuring Amide II bonding (p = 0.003) (Fig. 5b).

The in-between group comparisons of the absorbance spectra demonstrated significant differences for most comparisons, apart from, rather surprisingly, the comparison of the most extreme values (<10 mm with ≥15 mm). Most of the comparisons between the clinical groups of the proportional cervical length excised did not show a significant difference (Supplementary Figures 4 and 5).

The high variability of polypeptides featuring Amide I bonding appeared to be consistent across untreated, treated and healthy controls, suggesting it is unlikely to be due to human sampling error (either through collection of liquid-based cytological samples or acquisition of spectra), and may suggest that synthesis of these molecules is independent of disease status.

## Discussion

Local conservative treatment for CIN has been associated with significant adverse sequelae in subsequent pregnancies^8^,^9^,^10^,^11^. The frequency and severity of these effects seem to correlate directly to the radicality and depth of the treatment^8^,^12^.

The analysis of the biochemical fingerprints obtained from samples collected from the cervix before and 6 months post-treatment revealed that excision of cervical tissue and endocervical glands impact on the absorbance spectra that appeared to be significantly different after treatment. The significant increase in polypeptides in the post-treatment group, as evidenced by Amide I/II bonding, may be a response to localized injury caused by the excisional treatment. Normal wound healing is typified by three overlapping phases; the inflammatory phase, the proliferation phase and the maturation phase^20^,^21^. At around 30 days following the initial injury and when the wound is closed, the maturation phase begins and cellular activity diminishes, the number of blood vessels regress and collagen is remodeled from type III to type 1. Although previous work has shown that cervical regeneration is almost complete 6 months after excisional treatment^22^, our findings suggests that cellular function remains elevated at this time point. The presence of scar tissue at the site of injury may account for the increased polypeptides, since collagen is composed of three separate polypeptide chains^23^, and production is increased 2–3 times more in fibroblasts isolated from scar tissue than from normal tissue^24^. Despite this, a healed wound will only achieve a maximum of 80% of the tensile strength of normal epithelium. Further follow up may have shown the differences resolve with time or, the distinction is a true reflection of the newly formed epithelium capping the original wound. Additionally, the number of endocervical cells collected in post-LLETZ liquid-based cytology samples has been shown to be significantly decreased^25^. Therefore, any changes in biochemical function following treatment are likely to be a reflection of the cellular function largely associated with squamous cells.

When the spectra in healthy controls post-treatment were compared to healthy untreated controls, the differences remained significant, suggesting that it is the treatment rather than the excision of the disease that alters the biochemical balance within the cervix. Cervical regeneration is dependent upon the depth of excision, the percentage of cervix excised and/or the remaining cervical tissue immediately after treatment^13^. Our findings show that patients who were classified as cytologically free of cervical abnormality and HPV negative, remained biochemically distinct from healthy controls, with an increase in polypeptides (Amide II bonding) remaining elevated in the treated group, which may be due to the previously hypothesized causes.

When the absorbance spectra were assessed for different treatment cone depths and proportions of cervical lengths excised, we found no direct influence of the different clinical groups on the spectra, although the number of samples was small in each group. The mechanism behind the high variability associated with the production of polypeptides that appears to be independent of disease status is unknown, and may be a reflection genetic variation or other patient-specific characteristics.

It has not been possible to accurately calculate the number of cells and the thickness of the cellular layer and this is a limitation of this study. The size of the cell pellet varied from patient to patient, and similarly the number, dimensions and type of cells varied between slides. The number of cells collected by a typical traditional Pap smear is reportedly between 50,000 to 350,000 cells, and estimated to be almost doubled with liquid-based cytology (LBC) sampling techniques^26^. We used one out of the 20 ml of the LBC solution for our analysis and with an estimated number of cells between 5,000–35,000 cells, although the washing stages would likely have reduced the number further. Furthermore, the length and dimensions of squamous cervical cells varies even in healthy cells and the dimensions are reduced in dyskaryotic cells^27^. Future studies should sample a standard weight of cells from the original pellet and dispense the final suspension in 2 × 50 μl aliquots (one on top of the other) to further standardize the technique.

Several reports have assessed the impact of cervical excision on subsequent clinical reproductive outcomes; others suggested that it is the presence of the pre-cancer itself that also contributes to the adverse sequelae^28^. It is likely that excision of cervical tissue causes a disruption of the immune defense mechanisms, the natural production of antimicrobials and the mucus secretion from endocervical glands. These changes together with alterations caused by the disease are likely to interact with genetic, viral and microbial factors in a complex interplay within the vagina^29^,^30^.

This is the first report that assessed the impact that local excisional treatment for cervical pre-invasive disease has on the biochemical fingerprint and molecular processes within the cervix. We were able to show that excision causes major alterations in the cervico-vaginal environment and further research should establish the pathophysiological processes that may be correlated to adverse obstetric sequelae. We did not identify direct correlation of the changes to the radicality of the excision measured by cone depth and cervical proportion excised, but the numbers in each group were small and the results should be interpreted with caution. Further research should explore in more detail the impact of the severity/grade of CIN and/or presence of HPV infection on the biochemical spectra and should further describe the biochemical alterations in treated individuals with or without positive cytology and/or HPV infection post-treatment in larger cohort. This will allow a more comprehensive exploration of the impact of treatment; this was not feasible in this analysis as the number of samples in these subgroups was small for any valid comparisons.

The mechanism that accounts for the increased risk of second trimester loss and preterm birth associated with CIN and its treatment is not yet clarified. While acquired mechanical weakness of the cervix secondary to surgery might seem a logical assumption, more subtle mechanisms may be involved. Histological changes in the healed cervix affecting the tensile strength or changes in the innate immune system and vaginal microenvironment may also be involved. All these interactions may be best described in the absorbance spectra of the metabolic changes in the vagina as described in this study.

In conclusion, this study clearly demonstrates that local treatment for cervical pre-invasive disease has a direct impact on the biological and biochemical processes within the cervix, and this may correlate to the adverse sequelae described in future pregnancies that include preterm birth and premature rupture of the membranes, possibly as a result of ascending infections and disruption of the immune defense mechanisms. Correlation of the specific biochemical markers in the produced spectra with the outcomes of subsequent pregnancies in the future may allow the detection of women at high risk of preterm birth and enable the selection of women at high risk that would benefit from intensive antenatal surveillance when pregnant^31^. Furthermore, further exploration of the mechanistic aspects leading to these changes in the metabolic spectra may permit the use of more targeted cause-directed preventative treatment in the future.

## Materials and Methods

### Study population – Inclusion and Exclusion criteria

Ethical approval was obtained from the National Research Ethics Service Committee London - Fulham (Approval number 13/LO/0126). This study was conducted according to the principles of the Declaration of Helsinki and all other applicable national or local laws and regulations. All patients gave written informed consent before any protocol-specific procedure was performed.

We included pre-menopausal, non-pregnant women of reproductive age (18–45 years of age) who attended the colposcopy and were planned to undergo local cervical treatment at Imperial College NHS Healthcare Trust. We collected samples before treatment, and a repeat sample 6 months after treatment. We also recruited a population of women with normal cytology (+/− negative HPV DNA test) attending the colposcopy or general gynecology clinics that would serve as healthy controls. The samples for the healthy group were collected at one time point. The recruitment commenced in May 2013 and was completed in May 2015.

Women were included irrespective of their ethnicity, parity, smoking habits, phase in their cycle and use of contraception. The type of contraception and the time of their cycle (follicular or luteal) were documented. Women who were HIV or hepatitis B/C positive, women with autoimmune disorders, and women that received antibiotics or pessaries within 14 days of sampling were excluded. Detailed medical and gynecological history was collected for each patient including time since last sexual intercourse. Ethnicity was self-reported as Caucasian, Asian or Black.

Patients were anonymized and assigned a unique identifier. For each patient and visit, we collected data on the cytology, HPV DNA test and typing and histology, if available. The cytology result was classified as normal, borderline or mild dyskaryosis (low-grade squamous intraepithelial lesion [LSIL]), moderate or severe dyskaryosis (high-grade squamous intraepithelial lesions [HSIL]) and invasive cervical cancer (ICC). The histology was defined as normal, CIN1, CIN2, CIN3 or invasive cervical cancer.

Transvaginal ultrasound (Voluson E6 with a 5–9 MHz (RIC5-9-RS series) transvaginal probe (GE Healthcare, Zipf, Austria) was used to measure the cervical length and volume immediately prior to excision. The dimensions of the cervical cone were measured using electronic calipers. The volume was also determined by water displacement, using a 50 ml syringe. These measurements were taken prior to fixation in formaldehyde, which may result in a degree of sample shrinkage, and thus underestimate the size of the excised specimen. These data were used to calculate the proportion (percentage) of the length or volume excised. Patients included in the analysis of cone depth were categorized according to 1 of 4 categories (untreated healthy cervix; treated/cone depth: 1 = <10 mm, 2 = 10–14 mm, 3 = ≥15 mm). Patients included in the analysis of percentage excision were categorized according to 1 of 6 categories (untreated healthy cervix; treated/percentage excision: 1 = 0–10%, 2 = 11–20%, 3 = 21–30%, 4 = 31–40%, 5 = >40%).

### Sample collection and processing

A sterile, disposable speculum was inserted, without lubricant, and a cervical sample of ThinPrep, liquid-based cytology (LBC) was taken from the cervix (ThinPrep, HOLOGIC Inc., Bedford, USA). This was analyzed for cytological diagnosis and HPV DNA test and typing. HPV DNA test and 16/18 genotyping was carried out according to manufacturer’s guidelines using the Abbott RealTime High Risk (HR) HPV assay on Abbott M2000 platform; a clinically validated *in vitro* polymerase chain reaction (PCR) assay with identification of HPV-16, −18 or any other of 12 HR HPV subtypes (31, 33, 35, 39, 45, 51, 52, 56, 58, 59, 66, 68)^32^. From the remaining methanol-based fluid, 1 ml was stored at 4 °C and was used for biospectroscopy analysis at the Centre for Biophotonics, Lancaster University, England. Routine high vaginal microbiology swabs were taken and sent for microscopy, culture and sensitivity. These swabs were also used to diagnose bacterial vaginosis based on the Hay/Ison criteria^33^. Vaginal pH was measured using CarePlan VpH gloves (Inverness Medical, Unipath Ltd., Bedford, UK).

### Biospectroscopy: slide preparation

Each sample was agitated to disperse the cell pellet, and then a 500 μl aliquot collected into a clean micro tube. Samples were centrifuged at 2000 rpm for 5 minutes and the ThinPrep supernatant was aspirated to remove its spectral signature (i.e., the methanol fixative). Each sample was then immersed in 500 μl of distilled H_2_O, agitated and centrifuged again. The supernatant was removed again and the wash step repeated once more. The final pellet was immersed in 100 μl of distilled H_2_O, agitated and dispensed onto IR-reflective glass slides (Low-E; Kevley Technologies Inc., Chesterland, OH, USA). Whenever possible, the final suspension was dispensed onto the smallest area possible onto the slide (typically between 1–1.5 cm) and every effort was made to achieve a uniform spread of cells. An example of a typical prepared slide is presented in Supplementary Figure 6. A ‘halo’ of dispensed cells can be seen with a denser concentration of cells at the epicenter. Each slide was allowed to bench dry for a minimum of 24 hours. Samples were then stored in a desiccator for a minimum of 48 hours to remove any residual water before spectral analysis.

For those samples with a resultant poor spectral signal considered to be due to fewer cells on the slide, repeat samples were prepared using the remaining 500 μl of the original sample and prepared as described above. However, the final 100 μl solution was dispensed as 2 × 50 μl aliquots, with a 24-hour drying period in between each dispensing, and each aliquot being dispensed directly on top of the previous one to concentrate the available cells. These samples were then desiccated as described above. No cell thickness control was conducted other than visual inspection by eye.

### ATR-FTIR spectroscopy, computational and statistical analyses

#### Spectral acquisition

Spectra were acquired using a Tensor 27 FTIR spectrometer with a Helios ATR attachment (Bruker Optik GmbH). The instrument was set to take 32 scans at 8 cm^−1^ wavenumber spacing with 2× interferogram zero-filling. Before the spectra were taken, the crystal was cleaned with distilled H_2_O and inspected by video camera to be free of any contaminants. Prior to mounting each sample on the stage, a background spectrum was acquired to calculate any environmental contamination, such as ambient light. This background spectrum is automatically subtracted from the subsequent sample spectra via built-in software. Once the background spectrum had been collected, the sample slide was mounted and stage moved to bring the cervical cells in contact with the diamond. Ten, random sites on the slide were selected for spectral acquisition by visually monitoring the sample via video and seeking an area were adequate cell coverage could be confirmed. Good cell coverage directly affects the strength and quality of the signal at a particular site.

The length and dimensions of squamous cervical cells varies in normal, and in dyskaryotic cells^27^. Since the aperture of the sampling area is 250 × 250 μm, the number of cells involved in the sampling area has been estimated (Supplementary Table 3), but the number would have varied according to disease state/treatment, the size of the original cell pellet and the presence/absence of glandular cells in addition to squamous cells. For these reasons, an accurate estimate of the number of cells and the thickness of the cellular layer is not possible. Spectra were converted to absorbance by Bruker OPUS software (Bruker Inc., Billerica, MA, USA).

#### Pre-processing and multivariate analysis of spectra

Each spectrum file was loaded as an ‘OPUS binary format’ file type using MATLAB R2014a software (Mathworks Inc., Natick, MA, USA) together with the toolbox ‘IRootLab’ (http://trevisanj.github.io/irootlab/). Once loaded in MATLAB, this toolbox provides a ‘User Interface’ where spectra can be processed and features of interest constructed. Extensive tutorials to support the use of ‘IRootLab’ are available at the URL above.

The loaded data was saved to a text file and cleared from the toolbox command window. The saved text filed was opened in Excel and the data classed (e.g., as pre- or post-treatment), and then reloaded via the ‘objtool’ function in ‘IRootLab’. Spectra were cut to the ‘fingerprint region’ (1800–900 cm^−1^), Savitsky Golay differentiated to 1^st^ order and vector normalized. Combining vector normalization with differentiation is a typical pre-processing approach^34^. These datasets were used to extract the individual biomarkers of interest using the feature construction function within the ‘Apply new blocks/more actions’ menu.

Principal component analysis coupled to linear discriminant analysis (PCA-LDA) cascade was performed on the original pre-processed files (i.e., classed, cut, differentiated and normalized files). All programming within the toolbox is automatic with the exception of calculating the number of principal components (*n*_PCS_) for PCA, which is done by hard programming directly into MATLAB’s command window to guarantee a ratio of 20 between the number of spectra and the number of variables inputted into LDA, to avoid potential arbitrary class separation. An example of this code is ‘data_draw_pca_pareto(ds01_A_B_Paired_diff01_vector01, 20),’ where code prior to the brackets is standard for calculating the Pareto chart; the name of the file is in brackets, followed by the ratio). Typically, the number of PCs was either 9 or 10. The PCA-LDA output provided a ‘feature’ for each spectrum for each patient (i.e., 10 for each patient). An average of these 10 features was taken and used to conduct the statistical analyses.

Peaks found within the ‘biochemical fingerprint’ region contain features specific to biomarkers as shown in Supplementary Table 1^35^. Changes in the availability of these biomarkers will result in changes in peaks (absorbance), which can reveal patterns of intracellular change. We therefore extracted 7 wavenumbers associated with biologically important biomarkers using means, SD and multiple *t* tests (corrected for multiple *t* testing using the Holm-Sidak method) to produce a rate of change for each wavenumber. These were extracted from pre-processed data (i.e., classed, cut, 1^st^ order differentiated, vector normalized) at specified wavelengths within MATLAB.

#### Impact of treatment

Demographic data were summarized using RStudio Version 3.2.1 (RStudio: Integrated Development for R. RStudio, Inc., Boston, MA, USA). Differences in the patient characteristics between the compared groups were assessed using Fishers exact test (GraphPad Prism 6, [GraphPad Software Inc., La Jolla, CA, USA]). P-value < 0.05 was considered to be statistically significant.

We assessed the impact that local treatment may have on the cervix by comparing the cervical cell spectral absorbance of women with paired samples before and after treatment using means, standard deviations (SD) and a Student’s *t* test (Comparison 1). In order to control whether the observed differences were a result of the treatment itself or due to the removal of the disease (CIN), we performed an additional subgroup analysis to compare normal women post-treatment (negative cytology and negative HPV DNA test), to untreated normal controls (negative cytology and negative HPV DNA test). This was also done using means, SD and a Student’s *t* test (Comparison 2). We further assessed whether the changes in the biochemical fingerprint of the cervix correlated to the absolute cone depth (mm) and the proportion (percentage) of cervical length excised. We compared the different cone depth/proportions groups and also compared these with healthy controls with negative cytology irrespective of HPV status by means, SD and one-way ANOVA (Comparison 3). All statistical analyses of spectra were conducted using GraphPad Prism 6 (GraphPad Software Inc., La Jolla, CA, USA).

## Additional Information

**How to cite this article:** Halliwell, D. E. *et al*. Tracking the Impact of Excisional Cervical Treatment on the Cervix using Biospectroscopy. *Sci. Rep.* **6**, 38921; doi: 10.1038/srep38921 (2016).

**Publisher's note:** Springer Nature remains neutral with regard to jurisdictional claims in published maps and institutional affiliations.

## Supplementary Material

Supplementary Information

## Figures and Tables

**Table 1:** Patient characteristics.

Characteristics	Comparison 1: Treated women with paired samples		*P-value*	Comparison 2: Normal post-treatment vs. normal untreated controls		*P-value*	Comparison 3: Treated by cone depth/proportion vs. normal untreated controls		*P-value*
	Pre-treatment (n = 29)	Post treatment (n = 33)		Normal post-treatment, (Cytology –ve/HPV –ve) (n = 39)	Normal controls (Cytology –ve/HPV –ve) (n = 20)		Post treatment (n = 58)	Controls (Cytology -ve; HPV status ignored) (n = 27)	
**Age, years**			0.69			0.87			0.50
Mean (SD, range)	30.3 (5.0, 25–43)	30.8 (5.0, 25–43)		30.8 (4.5, 25–43)	30.6 (4.3, 24–37)		31.3 (4.4, 25–43)	30.0 (4.4, 22–37)	
**Ethnicity, n/N (%)**			1.00			0.12			0.24
Caucasian	22/29 (76)	25/33 (76)		30/39 (77)	11/20 (55)		46/58 (79)	18/27 (67)	
Asian	5/29 (17)	6/33 (18)		6/39 (15)	4/20 (20)		8/58 (14)	4/27 (15)	
Black	2/29 (7)	2/33 (6)		3/39 (8)	5/20 (25)		4/58 (7)	5/27 (18)	
**Smoking status, n/N (%)**			0.77			**0**.04*			0.18
Non-smoker	23/29 (79)	25/33 (76)		27/39 (69)	19/20 (95)		41/58 (71)	23/27 (85)	
Current smoker	6/29 (21)	8/33 (24)		12/39 (31)	1/20 (5)		17/58 (29)	4/27 (15)	
**Contraception, n/N (%)**			0.98			0.05			0.06
Nil	11/29 (37)	11/33 (33)		10/39 (25)	13/20 (65)		16/58 (28)	16/27 (59)	
Condoms	1/29 (4)	2/33 (6)		6/39 (15)	2/20 (10)		8/58 (14)	4/27 (15)	
COCP	14/29 (48)	17/33 (52)		20/39 (51)	3/20 (15)		28/58 (48)	4/27 (15)	
POP	2/29 (7)	2/33 (6)		1/39 (3)	1/20 (5)		2/58 (3)	1/27 (4)	
Implant	0/20 (0)	0/33 (0)		0/39 (0)	0/20 (0)		0/58 (0)	1/27 (4)	
Mirena IUS	1/29 (4)	1/33 (3)		1/39 (3)	1/20 (5)		2/58 (3)	1/27 (4)	
Copper IUD	0/29 (0)	0/33 (0)		0/39 (0)	0/20 (0)		1/58 (2)	0/27 (0)	
Vaginal ring	0/29 (0)	0/33 (0)		1/39 (3)	0/20 (0)		1/58 (2)	0/27 (0)	
**Parity, n/N (%)**			1.00			0.52			0.51
Nulliparous	21/29 (72)	24/33 (73)		32/39 (82)	15/20 (75)		45/58 (78)	22/27 (81)	
Parous	8/29 (28)	9/33 (27)		7/39 (18)	5/20 (25)		13/58 (22)	5/27 (19)	
**Time since last intercourse, n/N (%)**			0.71			0.28			0.31
>48 hours	26/29 (90)	28/33 (85)		34/39 (87)	15/20 (75)		52/58 (90)	22/27 (81)	
<48 hours	3/29 (10)	5/33 (15)		5/39 (13)	5/20 (25)		6/58 (10)	5/27 (19)	
**Phase of menstrual cycle, n/N (%)**			0.40			0.14			0.08
Luteal	15/29 (52)	18/33 (55)		21/39 (54)	6/20 (30)		29/58 (50)	7/27 (26)	
Follicular	13/29 (45)	11/33 (33)		14/39 (36)	9/20 (45)		23/58 (40)	14/27 (52)	
Unknown	1/29 (3)	4/33 (12)		4/39 (10)	5/20 (25)		6/58 (10)	6/27 (22)	
**Vaginal pH**			0.61			0.92			0.37
<4.5	12/29 (41)	13/33 (39)		16/39 (41)	9/20 (45)		24/58 (41)	15/27 (56)	
≥4.5	16/29 (55)	20/33 (61)		21/39 (54)	9/20 (45)		31/58 (53)	10/27 (37)	
Unknown	1/29 (4)	0/33 (0)		2/39 (5)	2/20 (10)		3/58 (6)	2/27 (7)	
**Bacterial vaginosis, n/N (%)**			1.00			0.13			0.16
No	27/29 (93)	31/33 (94)		35/39 (89)	15/20 (75)		52/58 (90)	21/27 (78)	
Yes	2/29 (7)	2/33 (6)		3/39 (8)	2/20 (10)		5/58 (8)	3/27 (11)	
Unknown	0/29 (0)	0/33 (0)		1/39 (3)	3/20 (15)		1/58 (2)	3/27 (11)	
**HPV DNA test, n/N (%)**			**0**.0001*			1.00			0.59
Negative	1/29 (4)	27/33 (82)		39/39 (100)	20/20 (100)		46/58 (79)	20/27 (74)	
Positive	28/29 (96)^a^	6/33 (18)		0/39 (0)	0/20 (0)		12/58 (21)	7/27 (26)^b^	

^a^Two patients with ‘unclear’ results were included in this group; ^b^ Two patients with ‘unknown’ results were included in this group. COCP: Combined oral contraceptive pill; HPV: Human Papillomavirus; HSIL: High-grade intraepithelial lesion; IUD: Intrauterine device; IUS: Intrauterine system; -ve: Negative; POP: Progesterone-only pill; SD: Standard deviation.
