# Supplementary material for: Tracking the Impact of Excisional Cervical Treatment on the Cervix using Biospectroscopy
Source: Sci Rep. 2016 Dec 15;6:38921. doi: 10.1038/srep38921 (PMC5156919; doi:10.1038/srep38921)
Supplement: Supplementary Information [file srep38921-s1.pdf]

Supplementary Information

Tracking the Impact of Excisional Cervical Treatment on the Cervix using Biospectroscopy

Diane E. Halliwell,[1] Maria Kyrgiou,[2,3] Anita Mitra,[2,3] Ilkka Kalliala,[2,3] Evangelos Paraskevaidis,[4] Georgios Theophilou,[5] Pierre L. Martin-Hirsch,[6] Francis L. Martin[1,7]

[1] Centre for Biophotonics, LEC, Lancaster University, Lancaster, UK; [2] Institute of Reproductive and Developmental Biology, Department of Surgery & Cancer, Faculty of Medicine, Imperial College, London, UK; [3] West London Gynaecological Cancer Centre, Imperial College NHS Healthcare, London, UK; [4] Department of Obstetrics and Gynaecology, University of Ioannina, Ioannina, Greece; [5] St James Hospital, Leeds, West Yorkshire, UK; [6] Department of Obstetrics and Gynaecology, Lancashire Teaching Hospitals NHS Trust Foundation, Preston, UK; [7] School of Pharmacy and Biomedical Sciences, University of Central Lancashire, Preston, UK

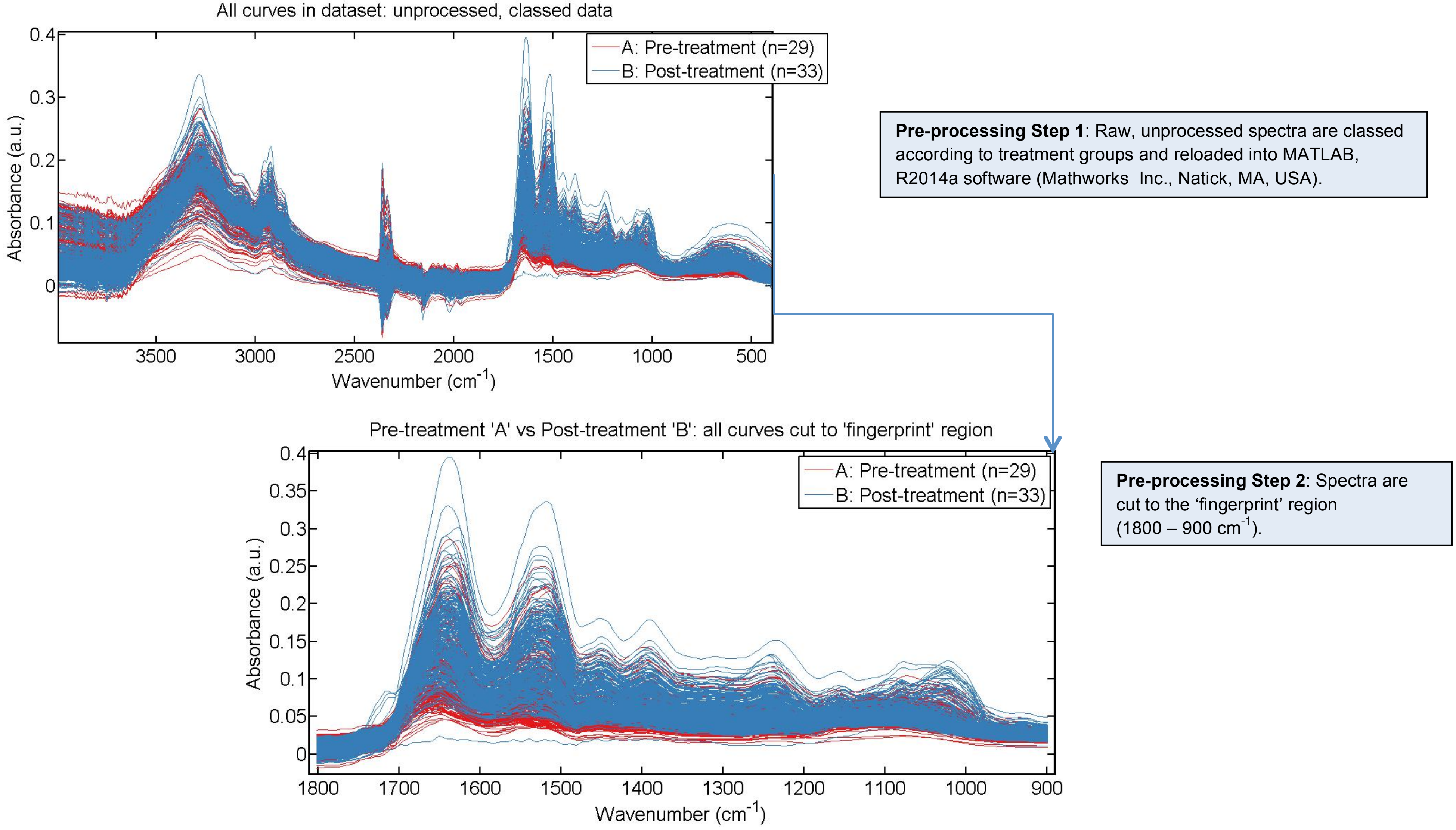

Supplementary Figure 1. Processing steps for spectra obtained using ATR-FTIR spectroscopy. Step 1: Classifying the data by treatment group; **Step 2:** Cutting the spectra to the fingerprint region (1800-900 cm^{-1}). ATR-FTIR: Attenuated total reflection Fourier-transform Infrared.

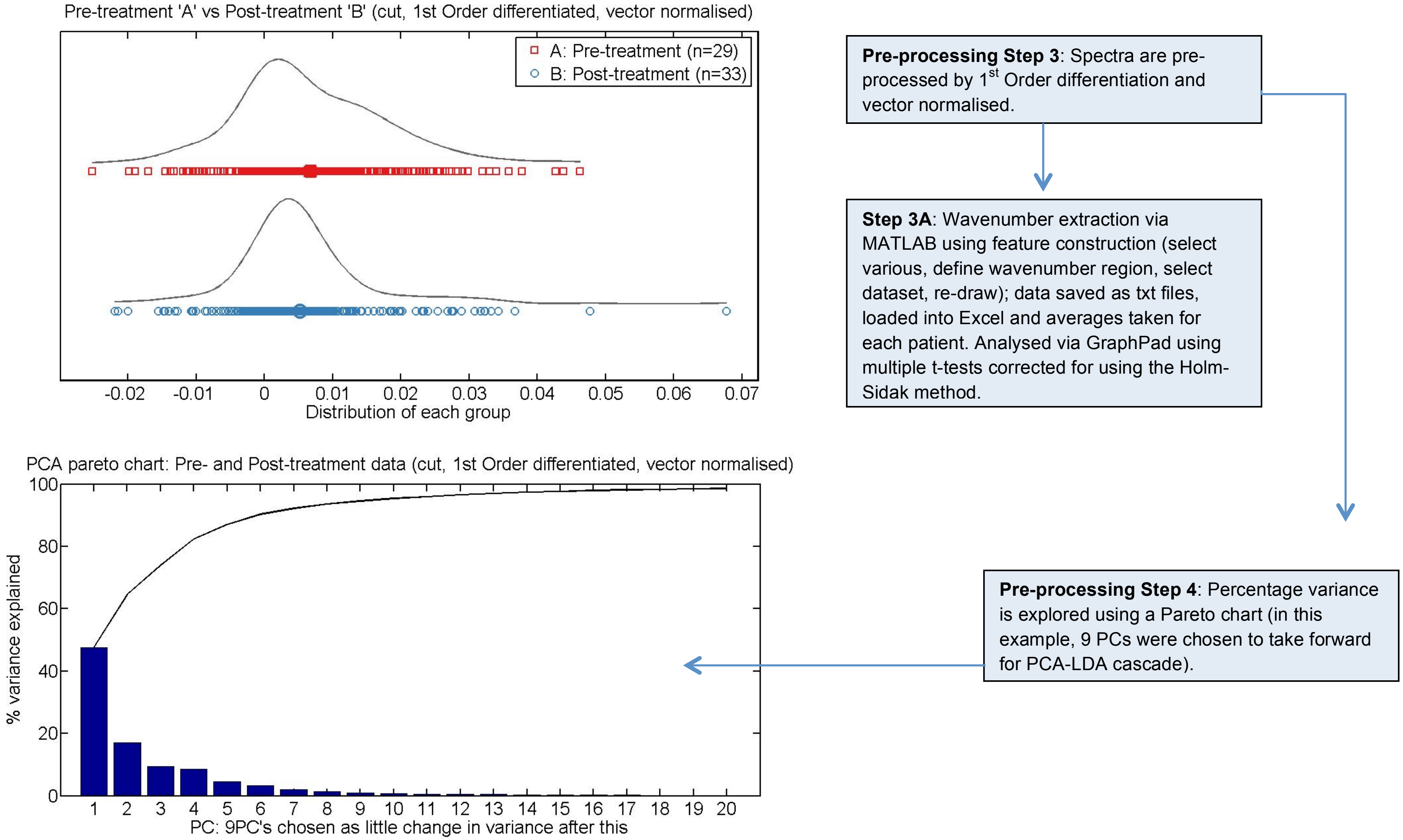

Supplementary Figure 2. Processing steps for spectra obtained using ATR-FTIR spectroscopy. Step 3: 1st order differentiated, followed by vector normalisation; **Step 3A:** Wavenumber extraction using previous data; **Step 4:** Calculation of percentage variance (i.e., number of Principal Components [PCs] to take forward for PCA-LDA cascade) using the Pareto chart. ATR-FTIR: Attenuated total reflection Fourier-transform Infrared. PCA-LDA: Principal Component Analysis coupled to Linear Discriminant Analysis.

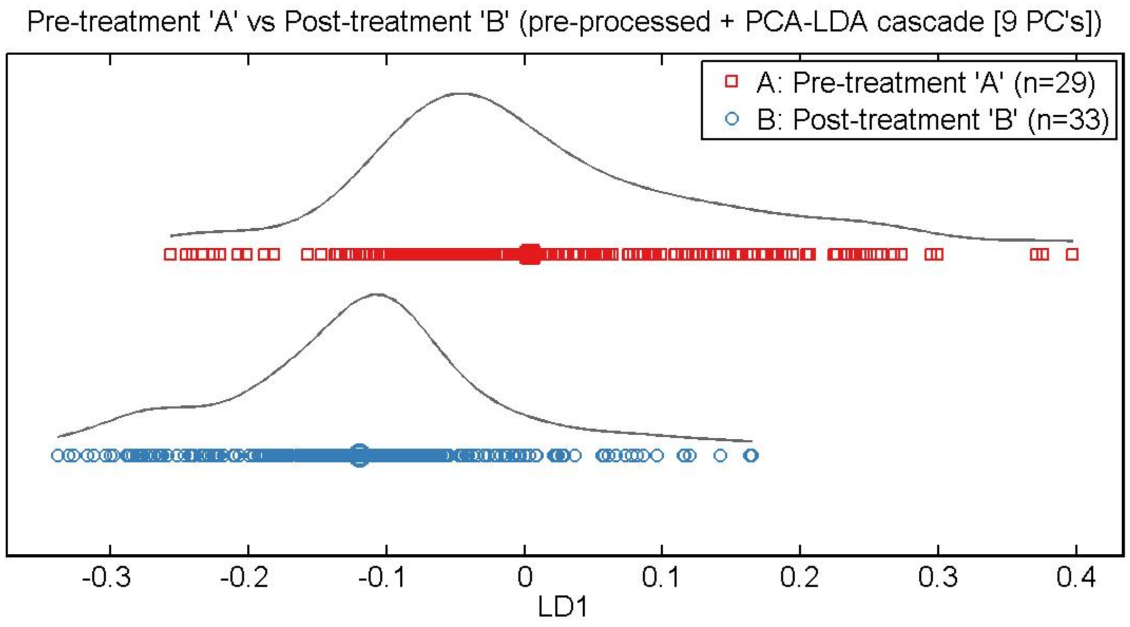

Step 5: PCA-LDA cascade applied using the number of PC's determined by Pareto.

Step 6: PCA-LDA output saved as txt file, loaded into Excel; averages taken for each patient and analysed via GraphPad Prism 6 (GraphPad Software Inc., La Jolla, CA, USA).

Supplementary Figure 3. Processing steps for spectra obtained using ATR-FTIR spectroscopy. Step 5: Applying PCA-LDA-Cascade using the previously defined number of principal component identified from Pareto plotting**.** ATR-FTIR: Attenuated total reflection Fourier-transform Infrared; LD: Linear Discriminant; PCA-LDA: Principal Component Analysis coupled to Linear Discriminant Analysis.

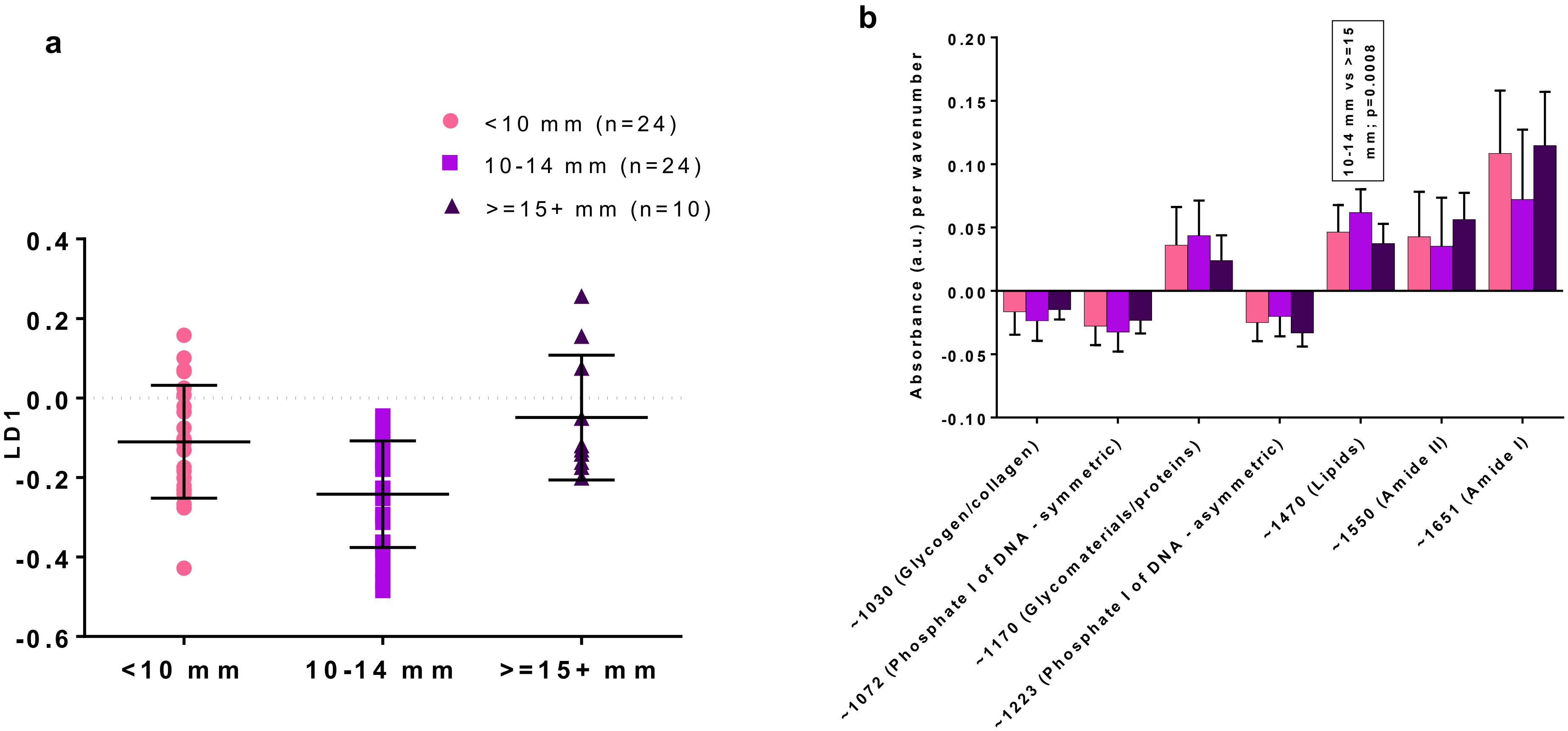

Supplementary Figure 4. PCA-LDA scores plot of ATR-FTIR spectra with regards to LD1: Cone depth (a) together with absorbance per wavenumber (b). Comparison of <10 mm with >=15 mm was not significantly different along LD1 (Mean/SD (**a**): -0.11/0.14 for <10 mm; -0.05/0.16 for >=15 mm; p=0.489, 95% CI = -0.19 to 0.07). Comparison of <10 mm with 10-14 mm was significantly different along LD1 (Mean/SD: -0.11/0.14 for <10 mm; -0.24/0.13 for 10-14 mm; p=0.006, 95% CI = 0.03 to 0.23). Comparison of 10-14 mm with >=15 mm was significantly different along LD1 (Mean/SD: -0.24/0.13 for 10-14 mm; -0.05/0.16 for >=15 mm; p=0.002, 95% CI = -0.32 to -0.06). Absorbance associated lipids were shown to have a significant positive rate of change for the 10-14 mm group compared with the >=15 mm group, evidencing their higher bioavailability. No significant differences were detected for wavenumbers associated with other biomarkers between all 3 groups **(b)**. ATR-FTIR: Attenuated total reflection Fourier-transform Infrared; CI: Confidence interval; SD: Standard deviation.

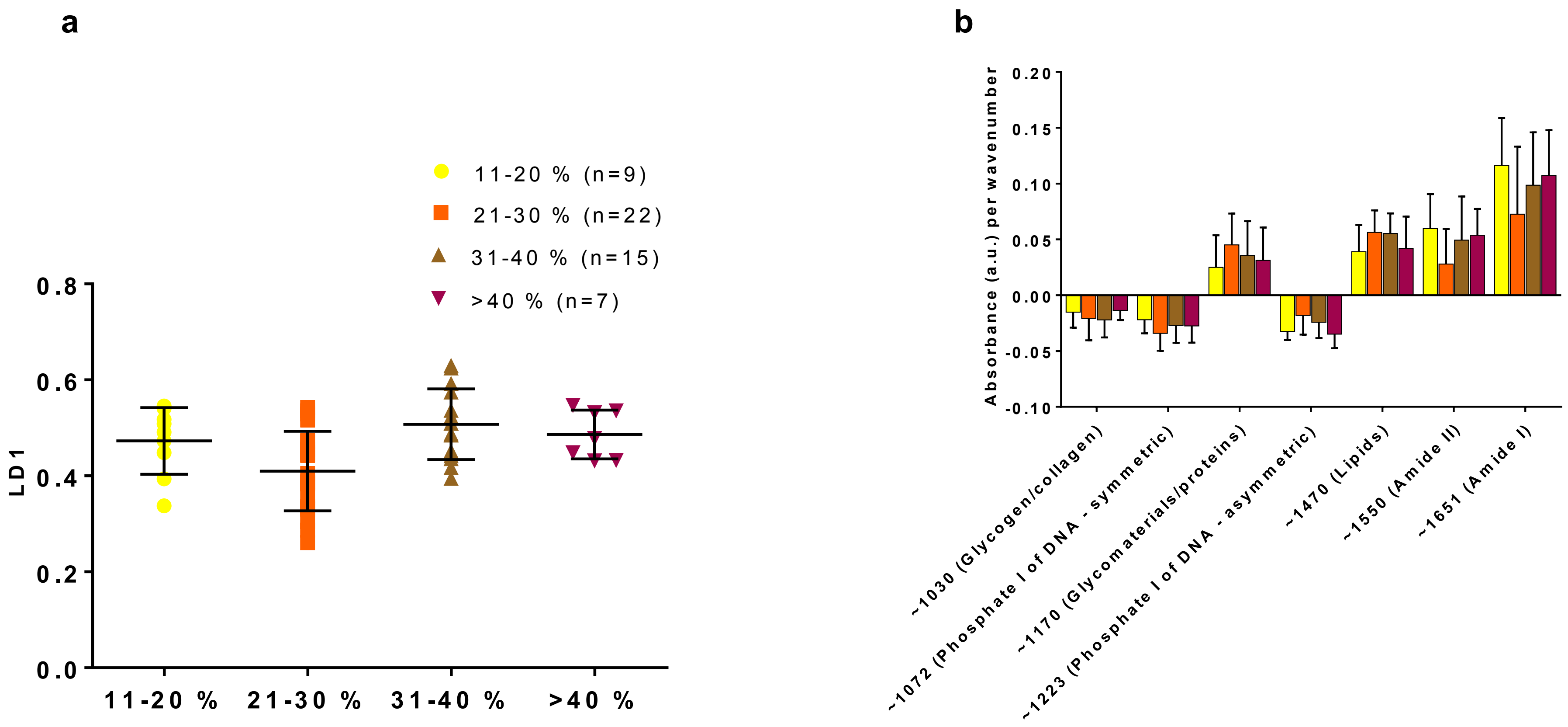

Supplementary Figure 5. PCA-LDA scores plot of ATR-FTIR spectra with regards to LD1: Percentage Excision (a) together with absorbance per wavenumber (b). Comparison of 11-20% with 21-30% was not significantly different along LD1 (Mean/SD (**a**): 0.47/0.07, 0.41/0.08 respectively; p=0.16; 95 CI -0.02 to 0.14). Comparison of 11-20% with 31-40% was not significantly different along LD1 (Mean/SD: 0.47/0.07, 0.51/0.07 respectively; p=0.69; -0.12 to 0.05). Comparison of 11-20% with >40% was not significantly different along LD1 (Mean/SD: 0.47/0.07, 0.49/0.05 respectively; p=0.98; 95 CI -0.11 to 0.09). Comparison of 21-30% with >40% was not significantly different along LD1 (Mean/SD: 0.41/0.08; 0.49/0.05 respectively, p=0.10; 95 CI -0.16 to 0.01). Comparison of 31-40% with >40% was not significantly different along LD1 (Mean/SD: 0.51/0.07, 0.49/0.05 respectively; p=0.92; CI -0.07 to 0.11). A significant difference was detected along LD1 for 21-30% vs 31-40% (Mean/SD: 0.41/0.08, 0.51/0.07, respectively; p=0.0016; 95 CI -0.16 to -0.03). No significant differences were detected for wavenumbers associated with the 7 biomarkers between all 4 groups (**b**). ATR-FTIR: Attenuated total reflection Fourier-transform Infrared; CI: Confidence interval; SD: Standard deviation.

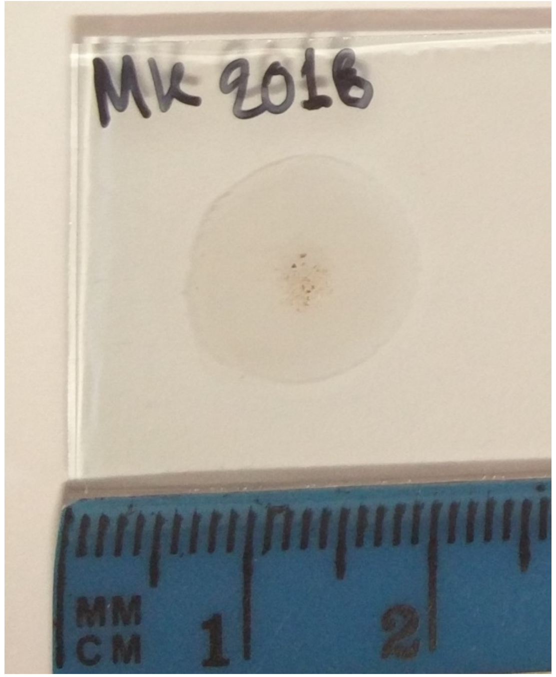

Supplementary Figure 6. Typical cervical cell preparation on Low –e slide. Typical dispensing involved the final washed pellet suspended in 100 µl of distilled water. The 'halo' of cells is estimated to be approximately 1.5 cm. Of note, is the denser concentration of cells at the centre of the dispensing area. Larger original cell pellets resulted in the expansion of the epicentre. Smaller pellets could result in fewer available cells over this area. To circumvent this, the final 100 µl aliquot was dispensed in 2 x 50 µl aliquots (the second being dispensed on top of the first), with a 24-hour drying period in between each dispensing.

Supplementary Table 1. Important biomarkers

**Tentative assignment of Biomarkers**[a]	**Wavenumber (cm^{-1})**
Amide I (of proteins predominantly in α helix conformation)	~1651
Amide II (of proteins predominantly in *β* sheet conformation)	~1550
Methylene chains in lipids	~1470
Phosphate I - asymmetric (DNA)	~1223
C-O bands from glycomaterials and proteins	~1170
Phosphate I - symmetric (DNA)	~1072
Glycogen & collagen	~1030

[a] Movasaghi *et al,* 2008[35]; N.B.: The signal at a particular wavenumber could have contributions from more than one biomarker.

Amide I and II are linked to the secondary structure of proteins and are indicative of their bioavailability.

Supplementary Table 2: Patient characteristics for the different cone depth and cervical proportion excised clinical groups (Comparison 3)

Characteristics	Normal controls (n=27)	Cone Depth[a] (n=58)			Percentage Excision[b] (n=53)			
		Category 1: <10 mm (n=24)	Category 2: 10-14 mm (n=24)	Category 3: >= 15 mm (n=10)	Category 2: 11-20% (n=9)	Category 3: 21-30% (n=22)	Category 4: 31-40% (n=15)	Category 5: >40% (n=7)
Age, years								
Mean (SD, range)	30.0 (4.4, 22-37)	30.6 (5.3, 25-42)	29.8 (3.1, 25-36)	33.5 (4.8, 25-43)	31.1 (4.3, 26-38)	30.3 (4.5, 25-42)	30.6 (5.2, 25-43)	33.0 (3.9, 25-38)
Ethnicity, n/N (%)								
Caucasian	18/27 (67)	19/24 (79)	20/24 (83)	7/10 (70)	5/9 (56)	19/22 (86)	14/14 (93)	6/7 (86)
Asian	4/27 (15)	4/24 (17)	3/24 (13)	1/10 (10)	4/9 (44)	2/22 (9)	0/15 (0)	0/7 (0)
Black	5/27 (18)	1/24 (4)	1/24 (4)	2/10 (20)	0/9 (0)	1/22 (5)	1/14 (7)	1/7 (14)
Smoking status, n/N (%)								
Non-smoker	23/27 (85)	19/24 (79)	15/24 (63)	7/10 (70)	7/9 (78)	17/22 (77)	8/15 (53)	5/7 (71)
Current smoker	4/27 (15)	5/24 (21)	9/24 (37)	3/10 (30)	2/9 (22)	5/22 (23)	7/15 (47)	2/7 (29)
Contraception, n/N (%)								
Nil	16/27 (59)	6/24 (25)	8/24 (34)	2/10 (20)	3/9 (33)	7/22 (32)	4/15 (26)	0/7 (0)
Condoms	4/27 (15)	4/24 (17)	2/24 (8)	2/10 (20)	1/9 (11)	2/22 (9)	3/15 (20)	2/7 (29)
COCP	4/27 (15)	12/24 (50)	12/24 (50)	4/10 (40)	5/9 (56)	12/22 (54)	6/15 (40)	4/7 (57)
POP	1/27 (4)	1/24 (4)	0/24 (0)	1/10 (10)	0/9 (0)	0/22 (0)	1/15 (7)	0/7 (0)
Implant	1/27 (4)	0/24 (0)	0/24 (0)	0/10 (0)	0/9 (0)	0/22 (0)	0/15 (0)	0/7 (0)
Mirena IUS	1/27 (4)	1/24 (4)	1/24 (4)	0/10 (0)	0/9 (0)	1/22 (5)	0/15 (0)	0/7 (0)
Copper IUD	0/27 (0)	0/24 (0)	0/24 (0)	1/10 (10)	0/9 (0)	0/22 (0)	0/15 (0)	1/7 (14)
Vaginal ring	0/27 (0)	0/24 (0)	1/24 (4)	0/10 (0)	0/9 (0)	0/22 (0)	1/15 (7)	0/7 (0)
Parity, n/N (%)								
Nulliparous	22/27 (81)	19/24 (79)	20/24 (83)	6/10 (60)	8/9 (89)	18/22 (82)	12/15 (80)	5/7 (71)
Parous	5/27 (19)	5/24 (21)	4/24 (17)	4/10 (40)	1/9 (11)	4/22 (18)	3/15 (20)	2/7 (29)
Time since last intercourse, n/N (%)								
>48 hours	22/27 (81)	19/24 (79)	24/24 (100)	9/10 (90)	6/9 (67)	21/22 (95)	15/15 (100)	7/7 (100)
<48 hours	5/27 (19)	5/24 (21)	0/24 (0)	1/10 (10)	3/9 (33)	1/22 (5)	0/15 (0)	0/7 (0)
Phase of menstrual cycle, n/N (%)								
Luteal	7/27 (26)	14/24 (58)	9/24 (38)	6/10 (60)	6/9 (67)	12/22 (55)	6/15 (40)	3/7 (43)
Follicular	14/27 (52)	8/24 (34)	12/12 (50)	3/10 (30)	3/9 (33)	10/22 (45)	6/15 (40)	3/7 (43)
Unknown	6/27 (22)	2/24 (8)	3/24 (12)	1/10 (10)	0/9 (0)	0/22 (0)	3/15 (20)	1/7 (14)
Vaginal pH								
<4.5	15/27 (56)	12/24 (50)	9/24 (38)	3/10 (30)	4/9 (44)	9/22 (41)	8/15 (53)	2/7 (29)
>=4.5	10/27 (37)	11/24 (46)	14/24 (58)	6/10 (60)	5/9 (56)	12/22 (55)	6/15 (40)	4/7 (57)
Unknown/NA	2/27 (7)	1/24 (4)	1/24 (4)	1/10 (10)	0/9 (0)	1/22 (4)	1/15 (7)	1/7 (14)
Bacterial vaginosis, n/N (%)								
No	21/27 (78)	22/24 (92)	22/24 (92)	8/10 (80)	8/9 (89)	22/22 (100)	14/15 (94)	5/7 (71)
Yes	3/27 (11)	2/24 (8)	1/24 (4)	2/10 (20)	1/9 (11)	0/22 (0)	0/15 (0)	2/7 (29)
Unknown	3/27 (11)	0/24 (0)	1/24 (4)	0/10 (0)	0/9 (0)	0/22 (0)	1/15 (6)	0/7 (0)
Follow up cytology/HPV, n/N (%)								
Normal & HPV -ve	NA	14/24 (58)	19/24 (79)	6/10 (60)	8/9 (89)	10/22 (45)	14/15 (93)	4/7 (58)
Normal & HPV +ve	NA	4/24 (17)	4/24 (17)	1/10 (10)	1/9 (11)	7/22 (32)	0/15 (0)	1/7 (14)
LSIL & HPV –ve	NA	3/24 (13)	1/24 (4)	2/10 (20)	0/9 (0)	3/22 (13)	1/15 (7)	1/7 (14)
LSIL & HPV +ve	NA	1/24 (4)	0/24 (0)	1/10 (10)	0/9 (0)	1/22 (5)	0/15 (0)	1/7 (14)

Characteristics	Normal controls (n=27)	Cone Depth[a] (n=58)			Percentage Excision[b] (n=53)			
		Category 1: <10 mm (n=24)	Category 2: 10-14 mm (n=24)	Category 3: >= 15 mm (n=10)	Category 2: 11-20% (n=9)	Category 3: 21-30% (n=22)	Category 4: 31-40% (n=15)	Category 5: >40% (n=7)
HSIL & HPV -ve	NA	1/24 (4)	0/24 (0)	0/10 (0)	0/9 (0)	1/22 (5)	0/15 (0)	0/7 (0)
HSIL & HPV +ve	NA	1/24 (4)	0/24 (0)	0/10 (0)	0/9 (0)	0/22 (0)	0/15 (0)	0/7 (0)
HPV DNA test, n/N (%)								
Negative	20/27 (74)	18/24 (75)	20/24 (83)	8/10 (80)	8/9 (89)	14/22 (64)	15/15 (100)	5/7 (72)
Positive	7/27 (26)[c]	6/24 (25)	4/24 (17)	2/10 (20)	1/9 (11)	8/22 (36)	0/15 (0)	2/7 (28)

[a] Patients were included in the dimensional analyses if they had had treatment and had follow up data at 6 months after treatment. [b] There was insufficient patients in Category 1 to include in the analysis.

[c] Patients with a HPV result of ‘unknown’ were included in the positive group. No segregation was made based on histology, cytology or HPV testing. COCP: Combined oral contraceptive pill: IUD: Intrauterine device; IUS: Intrauterine system; NA: Not applicable; -ve: Negative; POP: Progesterone-only pill; +ve: Positive; SD: Standard deviation.

Supplementary Table 3. Estimated length and height of squamous cervical cells

**Tissue type**[a]	**Length (μm)**	**Height (μm)**	**Average number of cells in the sampling area (250 x 250 μm) based on length**[b]
Normal	16 – 66 (SD 3.2 - 10)	14 – 17 (SD 4.7 – 5.1)	3906 - 947
CIN1	12 – 46 (SD 4.2 – 9.2)	13 – 16 (SD 1.3 - 3.8)	5208 - 1359
CIN2	12 – 38 (SD 1.6 – 7.1)	13 – 17 (SD 1.7 – 4.1)	5208 - 1645
CIN3	13 – 20 (SD 2.3 – 5.9)	13 – 16 (SD 1.5 – 3.2)	4808 - 3125

[a] Walker *et al,* 2003[27]. b Calculated by using the sampling area of 250 x 250 μm/cell length. The depth of the sampling area is unknown. CIN: Cervical intraepithelial neoplasia; SD: Standard deviation.
